# Anti-VEGF-Resistant Retinal Diseases: A Review of the Latest Treatment Options

**DOI:** 10.3390/cells10051049

**Published:** 2021-04-29

**Authors:** Josh O. Wallsh, Ron P. Gallemore

**Affiliations:** 1 Department of Ophthalmology, Albany Medical College, Albany, NY 12208, USA; jwallsh@hotmail.com; 2 Retina Macula Institute, Torrance, CA 90503, USA

**Keywords:** anti-VEGF, macular degeneration, diabetic retinopathy, resistant, retinal vein occlusion

## Abstract

Anti-vascular endothelial growth factor (anti-VEGF) therapy currently plays a central role in the treatment of numerous retinal diseases, most notably exudative age-related macular degeneration (eAMD), diabetic retinopathy and retinal vein occlusions. While offering significant functional and anatomic benefits in most patients, there exists a subset of 15–40% of eyes that fail to respond or only partially respond. For these cases, various treatment options have been explored with a range of outcomes. These options include steroid injections, laser treatment (both thermal therapy for retinal vascular diseases and photodynamic therapy for eAMD), abbreviated anti-VEGF treatment intervals, switching anti-VEGF agents and topical medications. In this article, we review the effectiveness of these treatment options along with a discussion of the current research into future directions for anti-VEGF-resistant eyes.

## 1. Introduction

Vascular endothelial growth factor (VEGF) is now known to play a central role in the vascular changes associated with the leading causes of blindness in developed countries, namely age-related macular degeneration (AMD), diabetic retinopathy and retinal vein occlusions (RVOs) [1,2,3]. Each of these disorders is associated with vascular leakage and proliferation which can lead to retinal edema, hemorrhaging and vision loss. The advent of anti-VEGF therapy for cancer, specifically bevacizumab (IVB; Avastin; Genentech; South San Francisco, CA, USA), led to the use of the same drug in exudative AMD (eAMD) and was found to be highly effective. In clinical trials, this agent and the more recently developed drugs ranibizumab (IVR; Lucentis; Genentech; South San Francisco, CA, USA), aflibercept (IVA; Eylea; Regeneron; Tarrytown, NY, USA) and brolucizumab (IVBr, Beovu; Novartis; Basel, Switzerland) have been shown to be able to reduce vascular leakage and proliferation and, in anywhere from 50–95% of eyes, improve vision [4,5,6,7,8]. There are, however, a small percentage of patients −20–40% in eAMD [4] and 15–20% in diabetic retinopathy [9,10]—who do not adequately or fully respond to anti-VEGF therapy and require alternate treatment strategies for their management. Alternatives include steroids, topical medications, laser treatments (thermal and photodynamic therapy) and, more recently, a host of other potential agents to effect improvement. Herein, we review the biological bases for failure to respond and the current and imminent therapies that may be utilized. While an exhaustive search of all the previously investigated therapeutic options was not performed, the following is a presentation of the selected literature illustrative of the available approaches.

## 2. Background

For most causes of vascular leakage, anti-VEGF therapy alone is sufficient to reduce leakage and restore vision. In the case of eAMD, randomized clinical trials of treatment naïve eyes found that treatments are effective at reducing leakage and improving vision in 59–68% of cases for IVB [11,12], 61–75% for IVR [6,12] and 79–83% for IVA [4,6]. It is the 20–40% of patients that may not respond and an additional subset of partial responders who require alternative treatment approaches for maximal therapeutic effect. Given the ambiguity in these terms, Amoaku et al. attempted to define non-responders and partial responders in eAMD based upon visual and anatomic responses. [13] Non-responders were defined as visual acuity decline of greater than 5 letters and/or optical coherence tomography (OCT) findings of unchanged or increased macular thickness and pigment epithelium detachment (PED). Partial responders have a visual acuity improvement of 1–5 letters from baseline and/or the following OCT findings: 25–75% decrease in macular thickness, persistence of subretinal or intraretinal fluid and/or new subretinal or intraretinal fluid. An additional group, poor responders, was also defined as visual acuity decrease of 0–4 letters from baseline and/or <25% decrease in macular thickness on OCT. In the case of diabetic retinopathy and RVOs, there has been less consistency regarding the definition of anti-VEGF resistance. For diabetic retinopathy—specifically diabetic macular edema (DME)—anti-VEGF resistance has been mainly defined based on OCT images either utilizing the percent decrease in macular thickness compared to pre-treatment (<25%) [14,15] or a threshold based on the macular thickness after a series of anti-VEGF injections (ranging from ≥225–300 µm) [15,16,17,18]. On the other hand, the definition of anti-VEGF resistance in eyes with RVOs has been based both on OCT (persistent edema or fluid) and/or visual acuity (no improvement) parameters [19,20,21]. For the purposes of the following review of treatment options, partial, poor and non-responders have all been termed anti-VEGF-resistant cases. The development of anti-VEGF resistance can occur following any number of injections, but it is important to evaluate all the relevant data prior to classifying a patient as “anti-VEGF resistant.” While the recurrence or persistence of intraretinal or subretinal fluid on OCT is important, if the visual acuity is maintaining or improving then the eye may not truly be anti-VEGF resistant.

**Figure 1:**
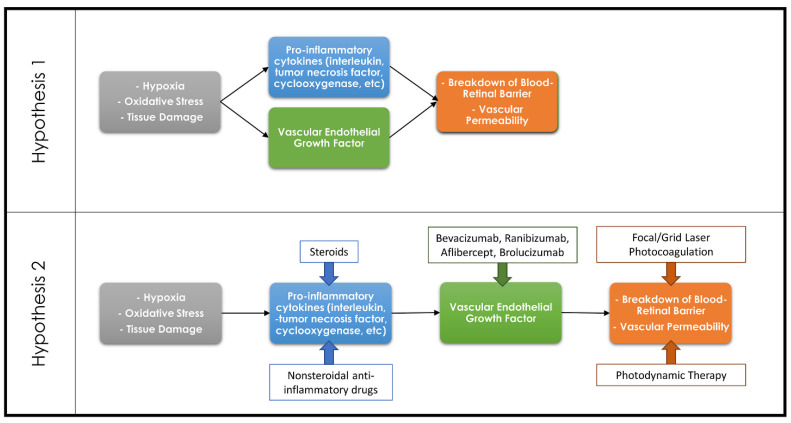
Diagram demonstrating the countering theories regarding the association of the inflammatory cascade and VEGF in the development of retinal pathologies. Hypothesis 1 theorizes that the inflammatory cascade and VEGF are both separate pathways resulting in a breakdown of the blood–retinal barrier, whereas Hypothesis 2 theorizes that the inflammatory cascade results in upregulation of VEGF which in turn leads to the breakdown of the blood–retinal barrier. Common therapeutic options and their targets within these pathways are demonstrated on the Hypothesis 2 diagram.

While the focus has been on VEGF-mediated leakage and vascular proliferation as the causes of vision loss in eAMD, diabetic retinopathy and RVOs, it is clear that there are other players involved. The literature is confusing, however, with some review articles talking about VEGF as being the corner stone or rate limiting factor causing pathologic vascular leakage and proliferation. Other reviews describe separate pathways where cytokines, for example, directly mediate leakage independent of VEGF (Figure 1) [22,23,24,25]. There is literature supporting both views—for example, the cytokine prostaglandin E2 can directly cause vascular leakage and proliferation but can also upregulate VEGF [26,27,28,29]. Both pathways likely play some role in the development of leakage.

Patients with anti-VEGF-resistant leakage have a different biological picture than the average person. Given the origins of anti-VEGF therapy are in oncology, resistance in cancers may provide insight into the culprits of anti-VEGF resistance in retinal diseases. These studies have demonstrated the importance of alternative proangiogenic factors in the development of anti-VEGF resistance such as platelet-derived growth factor, fibroblast growth factor, placental growth factor, interleukins and transforming growth factor-β [30,31]. Many of these factors have also been implicated in the development of retinal diseases and may be upregulated in light of VEGF blockade [32,33,34,35]. As such, there have been investigators promoting the sampling of factors in the vitreous fluid to develop customized approaches for the treatment of retinal diseases [36]. Until such therapeutic options are available, the following treatment approaches are capable of treating such anti-VEGF-resistant cases.

## 3. Exudative Age-Related Macular Degeneration

### 3.1. Alternative Anti-VEGF Dosing Strategies

The current anti-VEGF treatment strategy involves a loading dose series of three monthly injections followed by a transition to a pro re nata (PRN) or treat-and-extend strategy depending on physician preference. With all of these treatments, the shortest interval between injections is four weeks [37,38]. Several studies have evaluated shorter treatment intervals in anti-VEGF-resistant eAMD. Mimouni et al. retrospectively evaluated 27 eyes with anti-VEGF-resistant eAMD that received IVB every two weeks for a series of three to four injections. Overall, no significant change was appreciated in visual acuity or macular thickness, but a subset of 6 eyes (22.2%) were noted to have complete resolution of subretinal fluid on OCT [39]. Similarly, Witkin et al. retrospectively evaluated 18 eyes with anti-VEGF-resistant eAMD treated with alternating IVB and IVR every two weeks for four doses. These eyes experienced significant improvements in visual acuity (20/95 to 20/65) and macular thickness (455 to 387 µm) [40]. Mathematical models have also suggested similar intraocular benefits with the two-week regimen associated with more sustained anti-VEGF activity compared to four-week dosing [41]. In the case of IVA, the recommended dosing is a loading dose series of three injections every month followed by injections every eight weeks. Multiple studies have noted significant improvements in macular thickness when IVA was injected at four-week intervals, rather than every eight weeks, in anti-VEGF-resistant eyes. Visual acuity results, however, did not show a change when transitioned to such therapy [42,43]. These studies demonstrate that a subset of patients with eAMD initially resistant to anti-VEGF therapy may respond to shorter treatment intervals.

Treating at shorter intervals has not been the only alternative dosing strategy attempted in anti-VEGF-resistant eAMD. Some researchers have also attempted larger doses at regular intervals. Routine dosing of IVR and IVA have been with 0.5 mg and 2 mg, respectively. The HARBOR study demonstrated equivalent outcomes between 0.5 and 2 mg IVR dosing in treatment naïve eAMD eyes [44]. Assessment of 2 mg IVR in anti-VEGF-resistant eAMD eyes was performed in the SAVE trial. Following the initial three-month loading dose, 87 eyes were noted to have significant improvements in visual acuity (3.3 letter increase) and macular thickness (33.1 µm improvement) [45]. These results were noted to persist for one and two years while undergoing continued 2 mg IVR dosing using a PRN regimen [46,47]. Fung et al. performed a randomized controlled trial comparing 0.5 and 2 mg dosing in anti-VEGF-resistant eAMD eyes; however, these results were limited by a small sample size (7 eyes receiving 2 mg, 2 eyes receiving 0.5 mg). At the six-month follow-up, significant improvements were noted in visual acuity and macular thickness in the 2 mg cohort only; although at one year these improvements diminished slightly and were no longer significant [48]. You et al. retrospectively evaluated 4 mg IVA in anti-VEGF-resistant eyes and demonstrated a significant improvement in macular thickness but visual acuity was unchanged [49]. Although higher anti-VEGF doses did not appear to be beneficial to outcomes in treatment naïve eyes, there is evidence that some anti-VEGF-resistant eyes may improve in response to these dosing changes.

Important considerations when employing either shorter treatment intervals or higher anti-VEGF doses are the possible systemic side effects. There have been concerns raised about systemic exposure to anti-VEGF following intravitreal injections leading to increased rates of myocardial infarctions, cerebrovascular accidents or thromboembolic events [50,51]. Such systemic exposures would likely be increased with the alternative dosing regimens discussed above, but no increase in these complications were noted. In more recent years, real world research has questioned whether these systemic complications were more common with intravitreal anti-VEGF injections [52]. These systemic side effects should still be considered when employing these alternative dosing regimens especially in at risk groups.

### 3.2. Tachyphylaxis and Switching Anti-VEGF Medications

Many eyes with eAMD initially responsive to a single anti-VEGF agent will develop resistance or tachyphylaxis over repeated treatments. To counteract this resistance, numerous researchers have proposed switching agents to a different anti-VEGF which may offer improved responsiveness. Similarly, some eyes will be unresponsive to one anti-VEGF medication but when challenged with a different agent show a substantial response. In a study of 63 treatment naïve eyes with eAMD initiating anti-VEGF therapy, 14 eyes (22.2%) were unresponsive to initial anti-VEGF medication and 8 eyes (12.7%) developed tachyphylaxis in the one year of follow-up [53]. This rate of tachyphylaxis certainly increases as patients are treated for beyond one year.

The U.S. Food and Drug Administration (FDA) approval of IVA for the treatment of eAMD offered a novel anti-VEGF medication option after years of only having IVR and IVB available. This was especially important for the treatment of anti-VEGF unresponsive eyes and those with tachyphylaxis. Numerous investigators have evaluated its efficacy in these circumstances and all demonstrated some benefit [54,55,56,57,58,59,60,61,62]. Two meta-analyses attempted to evaluate the pooled data from these numerous studies. Seguin-Greenstein et al. noted that macular thickness improved significantly in all studies but reported only a nonsignificant improvement in visual acuity. Although, the analysis did reveal a significant improvement in visual acuity in the pooled prospective studies [63]. Similarly, Spooner et al. noted significant macular thickness improvements in all studies with nonsignificant visual acuity improvements. This analysis once again noted better visual acuity results in the pooled prospective studies but were only significant at the six-month follow-up [64]. Both of these studies only evaluated the response one year after switching medications to IVA, but a few other studies have investigated longer results. Jørstad et al. prospectively evaluated 50 eAMD eyes resistant to IVB and/or IVR transitioned to IVA. After one and two years, the macular thickness improved significantly; however, visual acuity results demonstrated less benefit. At the one-year follow-up visual acuity was unchanged from baseline and, in fact, decreased at the two-year follow-up (20/36 to 20/42). In addition, 5 eyes (10%) were switched from IVA treatment prior to the completion of the two-years study period due to a poor response [60]. In a separate study, Spooner et al. evaluated 39 eAMD eyes with 48 months of follow-up after switching to IVA. Both visual acuity and macular thickness significantly improved after one year, but while macular thickness improvement persisted throughout, visual acuity returned to baseline by 48 months [54]. Another important consideration in the decision to transition to IVA is the presence of a vascularized PED which can be a treatment challenge in eyes with eAMD. Multiple studies have demonstrated significant benefits to the use of IVA over other anti-VEGF agents with flattening of the vascularized PED and improved visual acuity results [65,66,67]. Furthermore, multiple investigators have demonstrated a beneficial anatomic response in such eyes resistant to IVB or IVR associated with stable visual acuity outcomes [68,69,70,71]. Transitioning to IVA offers anatomic benefits and, in many eyes, those benefits will equate to visual improvement as well. Over time, however, these visual benefits appear to be transient which may hasten a switch to an alternative therapeutic option [72].

When eyes with eAMD develop tachyphylaxis or are unresponsive to IVA, some have theorized a switch back to IVR (or even IVB) may be beneficial. The SAFARI study prospectively evaluated switching to IVR for a series of three monthly doses followed by PRN dosing for the six-month trial. A total of 100 eyes were included and nonsignificant improvements in mean macular thickness (−35.38 µm) and visual acuity (+1.9 letters) were noted at six months. In total, 34% of eyes were noted to have an increase of 5 or more letters after the switch in therapy [73]. Other smaller, retrospective studies have demonstrated similar benefits when switching to IVR in a subset of eyes with eAMD unresponsive to IVA [74,75]. In addition, the more recently FDA approved IVBr offers an additional therapeutic option for anti-VEGF-resistant eAMD that has developed tachyphylaxis to other agents. We reported in a case series of six anti-VEGF-resistant eyes the macular thickness benefit associated with a switch to IVBr, including eyes previously treated with IVA. These results did not demonstrate improved visual acuity outcomes [76].

The development of tachyphylaxis offers a treatment challenge, but numerous studies have demonstrated that switching anti-VEGF medication can be beneficial for many such eyes. Both IVA and IVBr appear to play a key role in the initial therapeutic approach when treating eyes that develop tachyphylaxis to other therapeutic agents. These agents have significantly different molecular structures and sizes when compared to the ranibizumab and bevacizumab compounds which may play a role when the retina becomes sensitized to these anti-VEGF treatments.

### 3.3. Photodynamic Therapy

Photodynamic therapy (PDT) as a treatment option for eAMD precedes the current anti-VEGF therapies but has fallen out of favor as a primary treatment. The therapy relies on an intravenous injection of verteporfin (Visudyne; Bausch + Lomb; Rochester, NY) which is then activated when in the retinal and choroidal circulation by a low-energy laser at 689 nm wavelength. Activation of verteporfin results in the creation of free radicals which cause vascular endothelial cell damage. When applied to the choroidal neovascular membrane in eAMD, it results in regression of the membrane but recurrence is common and repeat treatment is required. In eyes with anti-VEGF resistance, PDT may offer an alternative treatment option.

Multiple studies have evaluated various PDT protocols in the treatment of anti-VEGF-resistant eAMD. Park et al. retrospectively evaluated 78 eyes with anti-VEGF-resistant eAMD treated with reduced fluence PDT (laser delivered for a shorter period of time resulting in a 20% reduction in energy density delivered from standard therapy) [77]. All enrolled eyes initially received PDT as “rescue therapy” based on a lack of resolution of subretinal and/or intraretinal fluid along with macular thickening despite a series of three anti-VEGF injections. Anti-VEGF injections were allowed to be initiated on a PRN basis three months after PDT treatment and repeat “rescue therapy” with PDT allowed if the same parameters previously discussed were met again. Three months after PDT, visual acuity improved significantly but subsequently decreased and was significantly worse than baseline from 24 through 60 months. In addition, exudation as determined by OCT had resolved in 55 eyes (77%) after three months but at about a year had recurred in 49 of these eyes. During the follow-up, 34 patients (44%) required repeat PDT “rescue therapy” with macular atrophy overall reported as the most common complication in 30 patients (39%) which may explain the poorer visual outcomes. Two other studies investigated combination PDT and anti-VEGF treatment (double therapy) in eAMD eyes resistant to anti-VEGF monotherapy. Tozer et al. combined half-fluence PDT (defined as laser applied at 300 mW, 25 J, 83 s) with IVB in 26 eyes. These eyes were followed for 6 months after combination therapy and anti-VEGF injections were continued on a PRN basis after combination therapy. Visual acuity improved significantly at three months and macular thickness significantly improved at all follow-ups [78]. Silva-Garcia et al. also treated anti-VEGF-resistant eAMD eyes with double therapy and noted relative stability five years after treatment in 11 eyes with many others remaining inactive throughout that time period. An important note regarding the 29 eyes included in this study, the inclusion criteria required inactive choroidal neovascularization for one year following combination treatment so not all eyes treated at this practice were included [79]. Triple therapy (PDT, intravitreal anti-VEGF and intravitreal steroids) has not been studied specifically in this population of anti-VEGF-resistant patients, but may offer an additional benefit to these challenging cases providing treatment of multiple aspects associated with the pathophysiology of eAMD [80,81]. The PDT to regress the choroidal neovascular membrane and vascular permeability, anti-VEGF treatment to counteract the drive for neovascularization and the anti-inflammatory properties of the steroid. Overall, PDT offers an effective therapy with an extended course of action; however, recurrence commonly occurs after a year so repeat therapy or an alternative treatment modality is necessary. Additionally, the concern exists for possible retinal pigment epithelium tears and progression of atrophy, so this must be considered and discussed when recommending such intervention.

### 3.4. Steroids

Intravitreal steroids have been a mainstay of therapy for numerous retinal vascular disorders for many years but have overall not found favor in the treatment of eAMD. In the case of anti-VEGF-resistant eAMD, some have proposed that an inflammatory component is partially to blame for this resistance [82]. This has led to the application of intravitreal steroids in the treatment of anti-VEGF-resistant eAMD. Tao and Jonas evaluated combination intravitreal triamcinolone acetonide (IVTA; TRIESENCE; Alcon; Fort Worth, TX, USA) and IVB injections in 31 eyes with anti-VEGF-resistant eAMD [83]. These eyes were followed for seven months, receiving one combination treatment followed by two additional IVB injections every two months. Initially, visual acuity and macular thickness improved significantly at two and four months after combination therapy. At seven months, both visual acuity and macular thickness continued to be improved from baseline but no longer significantly. The intravitreal dexamethasone implant (DEX; Ozurdex; Allergan; Dublin, Ireland) offers an extended-release intraocular steroid therapy which has been evaluated by multiple investigators in combination therapy for anti-VEGF-resistant eAMD. Todorich et al. retrospectively evaluated 18 eyes over six months initially treated with a combination of intravitreal DEX and anti-VEGF followed by PRN anti-VEGF dosing. These eyes had a significant improvement in macular thickness but no significant change in visual acuity after six months [84]. Barikian et al. similarly evaluated 19 eyes with eAMD unresponsive to a series of IVB and IVR subsequently treated with a single combination treatment of intravitreal DEX and IVR followed by PRN IVR for six months. Again, a significant improvement in macular thickness was noted, but visual acuity remained unchanged [85]. Giancipoli et al. found similar anatomic benefit with intravitreal DEX in 11 eyes with eAMD resistant to anti-VEGF but without significant visual acuity improvement [86]. Intraocular pressure and cataract progression remain possible complications with such therapy and should be considered when initiating treatment with steroids. Although treatment with steroids may offer some benefit in anti-VEGF-resistant eAMD, the response has been short-lived and overall underwhelming.

### 3.5. Miscellaneous Treatment Considerations

In a number of smaller studies, alternative treatment approaches have been evaluated including the use of oral medications or surgical interventions (Table 1). Another important consideration is ensuring that the correct diagnosis is being treated when a patient is unresponsive or poorly responsive to anti-VEGF therapy. Specifically, in the case of eAMD, the possibility of polypoidal choroidal vasculopathy (PCV) should be considered.

Perceived to be a variant of eAMD, PCV is most commonly seen in Asian patients [87,88] with characteristic indocyanine green angiography findings. [89] Treatment of PCV can be challenging and numerous studies have evaluated possible treatment approaches including the use of anti-VEGF and PDT monotherapy. [90,91,92] Despite benefits to both therapeutic options alone, multiple studies have noted superior visual results with combination intravitreal anti-VEGF and PDT [89,93]. In addition, the rate of anti-VEGF resistance has been higher in PCV than in other eyes with eAMD which may partially explain the superior visual response of PCV to combination therapy [94,95]. The rate of anti-VEGF resistance and significant benefit with the addition of PDT are reasons to consider the possibility of PCV in eyes diagnosed with eAMD that do not respond to initial intravitreal anti-VEGF treatment.

## 4. Diabetic Retinopathy and Diabetic Macular Edema

### 4.1. Steroids

Intravitreal steroids have served an important role in the treatment of diabetic macular edema (DME) for many years, preceding the use of intravitreal anti-VEGF injections by a number of years [104,105,106]. The initial research utilized the IVTA injection, but in more recent years the availability of the DEX implant has provided a longer-term option for steroid therapy. In the treatment of DME, steroids act as an anti-inflammatory which helps to downregulate both pro-inflammatory and pro-angiogenic mediators in the eye that are paramount to the development of edema. As was the case for eAMD, intravitreal anti-VEGF therapy remains the standard of care for DME; however, many have utilized intravitreal steroid therapy to treat those who are resistant to such treatment.

Multiple investigators have evaluated both IVTA and DEX in the treatment of anti-VEGF-resistant DME. Kim et al. retrospectively evaluated 20 eyes with anti-VEGF-resistant DME treated with a single IVTA injection. These eyes experienced a significant improvement in both macular thickness and visual acuity at three months after treatment [107]. Jeon and Lee prospectively evaluated 20 eyes with anti-VEGF-resistant DME treated with a single IVTA and followed for three months after treatment. Their results showed significant improvements in both macular thickness and visual acuity at one and two months; however, the visual acuity improvement diminished at three months. Visual acuity improved from 47.1 to 53.3 letters at two months but decreased to 50.9 letters at three months [108]. Both of these studies support the benefit of IVTA as an alternative for anti-VEGF-resistant eyes, but not unexpectedly, repeated therapy is likely necessary. In the case of steroid therapy, repeated intravitreal injections do carry the additional risks of glaucoma and cataract progression [109,110].

The FDA approval of DEX for the treatment of DME offered another intravitreal steroid alternative to IVTA with the benefit of an extended therapeutic window. Multiple studies have investigated the use of DEX in the treatment of anti-VEGF-resistant DME. In several retrospective studies of anti-VEGF-resistant eyes, a single DEX implant was noted to improve both macular thickness and visual acuity for three months following treatment [16,111,112,113,114,115,116]. The visual acuity improvement waned by six months in those studies with sufficiently long follow-up [111,112]. Hatz et al. retrospectively evaluated two DEX implants separated by an average of four months in anti-VEGF-resistant DME with significant improvements in visual acuity and macular thickness noted [117]. Iacono et al. also evaluated anti-VEGF-resistant DME in 13 eyes treated with PRN DEX over one year of follow-up with significant improvements in visual acuity and macular thickness observed [118]. In the only prospective study of DEX implant monotherapy in anti-VEGF-resistant DME, Lazic et al. treated 16 eyes with a single DEX implant and reported significant improvements in macular thickness at one, two and three months. Visual acuity improved at one, two and three months as well but only significantly at two months (baseline: 20/69, one month: 20/58, two months: 20/51, three months: 20/53) [18]. Busch et al. performed the only comparative trial of DEX implants versus continued intravitreal anti-VEGF therapy in eyes with DME and an initial suboptimal response to anti-VEGF treatment. A total of 87 eyes were included with 44 eyes treated with anti-VEGF monotherapy throughout, 29 switched to DEX implant after a poor initial response to the loading dose and 14 switched after one year of anti-VEGF monotherapy. The eyes treated with DEX implants were also treated with anti-VEGF therapy concurrently and received between one to three DEX implants annually. At the culmination of the two years of follow-up, both the early and late eyes switched to the DEX implant demonstrated improvements in visual acuity and macular thickness greater than those appreciated in the anti-VEGF monotherapy group [119]. These studies overwhelmingly demonstrate the benefits of DEX implants in eyes with DME resistant to anti-VEGF therapy alone. Despite all of this strong evidence, the only randomized controlled trial with the DEX implant in anti-VEGF-resistant DME did not have such overwhelmingly positive results. Over 24 weeks, 65 eyes treated with the DEX implant and PRN IVR were compared to 51 eyes receiving PRN IVR monotherapy. There was a significantly greater improvement in macular thickness in the combination group but no difference in the improvement in visual acuity across cohorts. An important note, however, is that the combination group had a much higher percentage of patients experiencing large improvements in vision with ≥10 letters of improvement noted in 33% of the combination treatment eyes compared to just 16% of those treated with monotherapy. The poor mean visual acuity response in the combination group may be, in part, due to cataract progression resulting in significant vision loss (7 of 8 eyes with a ≥2 line decrease were phakic in the combination group) [120]. Patients with anti-VEGF-resistant DME appear to benefit from the addition of the DEX implant [121], but given the need for repeated treatments every four to six months, its greatest benefit is likely as an adjunctive therapy with intravitreal anti-VEGF injections.

There are two other extended-release steroid implants available that utilize fluocinolone acetonide (FA). The 0.59 mg intravitreal FA implant (Retisert; Bausch + Lomb; Rochester, NY, USA) requires surgical implantation but can provided years of therapy, whereas the more recently developed 0.19 mg intravitreal FA implant (Iluvien; Alimera Sciences Inc; Alpharetta, GA, USA) is an injectable implant that can be performed in clinic which can also last for a year or longer. Multiple studies have investigated the intravitreal FA implant in the treatment of anti-VEGF-resistant DME with beneficial, sustained visual acuity and macular thickness results [122,123,124,125,126,127,128]. Compared to the DEX implant, the 0.19 mg intravitreal FA implant offers continuous steroid therapy for longer treatment intervals (about 4 months compared to 8–24 months). Similar to previously discussed intravitreal steroid treatments, the FA implant appears to be most effective when supplemented with continued intravitreal anti-VEGF injections.

As an alternative to intravitreal steroid therapy, posterior subtenon triamcinolone has also been utilized in the treatment of DME with mixed results [129,130]. Eriş et al. retrospectively compared the treatment of anti-VEGF-resistant DME with combination posterior subtenon triamcinolone and intravitreal anti-VEGF versus anti-VEGF monotherapy. The 38 eyes treated with combination therapy (including a single posterior subtenon triamcinolone and PRN anti-VEGF) achieved a significant improvement in visual acuity and macular thickness after six months. The 34 eyes treated with PRN anti-VEGF as monotherapy were noted to have nonsignificant improvements in both measures [131]. There has been some evidence that the posterior subtenon application of steroids results in less steroid responsive intraocular pressure elevation than intravitreal steroids [132,133]. Although the research is limited to this single study, the use of posterior subtenon triamcinolone may be a useful adjunctive therapy in anti-VEGF-resistant DME if concern exists for steroid induced glaucoma.

### 4.2. Laser Photocoagulation

The use of thermal laser photocoagulation has been around for decades in the treatment of retinal diseases, especially vascular disorders such as diabetic retinopathy. Various forms of thermal laser photocoagulation have been utilized to treat different aspects of these diseases. Panretinal photocoagulation (PRP) applies equally spaced laser burns to the retinal periphery in eyes with proliferative diabetic retinopathy (PDR). These are applied to ischemic areas of the retina which effectively destroys those areas and halts the eyes’ drive to produce VEGF. Focal laser photocoagulation applies gentler, lower energy laser to leaking macular microaneurysms which are key to the development of DME. Grid laser photocoagulation similarly applies gentler, lower energy laser to diffuse areas of macular leakage instead of specifically targeting leaking microaneurysms. Finally, the more recently developed subthreshold or micropulse laser provides short, repeated bursts of laser energy which allows the tissue to cool prior to the application of further laser energy. This is different to traditional laser photocoagulation which provides a continuous wave of laser energy without requiring a cooling period between applications and thus limits the spread of laser damage to the surrounding structures. Micropulse laser has been utilized similarly to grid laser photocoagulation in the treatment of DME.

In eyes with PDR, multiple studies have shown better visual acuity benefits from the use of intravitreal anti-VEGF therapy over PRP treatment [7,134]. As discussed above, the driving force for the development of PDR is VEGF which results in retinal neovascularization. Regular application of intravitreal anti-VEGF injections can keep this at bay. A significant concern in the treatment of PDR, however, is the potential for catastrophic progression and profound vision loss in the absence of regular follow-up with the development of vitreous hemorrhage or tractional retinal detachment. Intravitreal anti-VEGF injections rely upon regular treatment to keep the neovascularization of PDR under control; thus, it is not a surprise that studies have shown significantly worse outcomes in PDR eyes lost to follow-up treated with anti-VEGF compared to PRP [135]. Hence, despite studies showing improved visual results with intravitreal anti-VEGFs, the use of PRP still remains commonplace and would be an obvious choice in an eye with anti-VEGF-resistant PDR.

The initial Early Treatment Diabetic Retinopathy Study demonstrated the benefit of laser photocoagulation in the treatment of clinically significant macular edema [136]. As has been the case for many of the previously discussed treatment modalities, it has largely been replaced by intravitreal anti-VEGF therapy as first line therapy for DME. While there has not been any studies to evaluate the use of focal or grid laser photocoagulation in the treatment of anti-VEGF-resistant DME, numerous studies have evaluated its use in combination with anti-VEGF therapy [137,138,139,140]. Although these results have not demonstrated improved outcomes with combination treatment compared to intravitreal anti-VEGF monotherapy, there has been some evidence of a decreased anti-VEGF burden with combination therapy [137,138]. These findings support a theoretical benefit of focal or grid laser photocoagulation in anti-VEGF-resistant DME.

As previously noted, micropulse laser offers an approach to the treatment of DME similar to grid laser photocoagulation with less collateral damage to the precious macular tissue. Multiple studies have demonstrated a significant decrease in the number of intravitreal anti-VEGF injections when combined with micropulse therapy to treat DME [141,142]. In the only study evaluating micropulse laser in anti-VEGF-resistant DME, 21 patients had one eye randomly assigned to micropulse laser with intravitreal anti-VEGF and the other eye treated with anti-VEGF monotherapy. At three months, visual acuity decreased significantly in the anti-VEGF monotherapy group (20/100 to 20/123) and increased significantly in the combination group (20/129 to 20/83) [143]. This therapy is a relatively recent advancement; hence, there is only limited research and long-term outcomes are unclear. Although, these results provide reason for optimism about its benefit in anti-VEGF-resistant DME.

Overall, laser photocoagulation, in all of its forms, has an important role in the treatment of diabetic retinopathy and DME especially as an alternative to intravitreal anti-VEGF in eyes that are resistant.

### 4.3. Miscellaneous Treatment Considerations

As discussed with anti-VEGF-resistant eAMD, some cases will have improved responses to a switch in anti-VEGF agent. This possibility has also been evaluated in anti-VEGF-resistant DME. Multiple studies have investigated switching from IVR and/or IVB to IVA in eyes with anti-VEGF-resistant DME. These studies have demonstrated significant improvements in visual acuity and macular thickness with this switch while utilizing either a standard every eight week or PRN dosing regimen [144,145,146].

The topical application of medication provides an additional therapeutic route in these anti-VEGF-resistant eyes. Various studies have evaluated the efficacy of topical anti-glaucoma medications as an adjuvant therapy to intravitreal anti-VEGF injections with mixed results. In a randomized controlled trial, the carbonic anhydrase inhibitor (CAI), dorzolamide (Merck & Co; Kenilworth, NJ, USA), was applied to one eye of 16 patients receiving monthly IVB in both eyes for DME. Comparison of the eyes treated with and without dorzolamide at three months did not demonstrate a difference in visual acuity or macular thickness results [147]. On the other hand, a similar study with topical dorzolamide-timolol (Akorn; Lake Forest, IL, USA) in 11 patients demonstrated significantly better visual acuity and macular thickness after three months in the eyes receiving topical treatment [147]. Multiple theories exist as to why such medications may provide improved results in combination with anti-VEGF therapy. Topical dorzolamide as well as oral CAIs have found utility in the treatment of other causes of macular edema such as retinitis pigmentosa [148,149]. Rat models have found carbonic anhydrase enzymes on the cell membranes of Muller cells and the retinal pigment epithelium [150]. Thus, it is possible that the dehydrating capacity of CAIs has activity on these retinal cells resulting in decreased macular edema. The second component of this combination drop, timolol, is a beta blocker which has been theorized to play a role in the downregulation of VEGF [151]. It appears that this combination therapy plays a synergistic role with intravitreal anti-VEGF therapy to accentuate its efficacy. Although studies have not evaluated the benefit of topical anti-glaucoma medications in anti-VEGF-resistant DME, these results appear to support its utility in such situations.

Another commonly used topical medication in the treatment of retinal diseases are nonsteroidal anti-inflammatory drugs (NSAIDs). We have previously reported of a single case of DME responsive to combination topical NSAID, steroid and CAI and subsequently maintained on the topical NSAID alone for a year [152]. In a cohort of 17 eyes with treatment naïve DME, Pinna et al. found significant improvements in macular thickness after administration of topical bromfenac (Bausch + Lomb; Rochester, NY, USA) twice daily, but no significant change noted in visual acuity [153]. Multiple studies have also evaluated the use of intravitreal NSAIDs in the form of diclofenac (Troge Medical GMBH, Hamburg, Germany) compared to and in combination with IVB. Overall, these studies have shown visual and macular thickness benefits to such treatment which, in some cases, was superior to IVB monotherapy [154,155,156]. These studies were all performed in treatment naïve eyes, but the evidence of the benefit of NSAID therapy as a possible adjuvant treatment with anti-VEGF injections appears consistent. Topical application of such medications comes with less significant side effects especially in the short term, most concerning corneal toxicity, so a trial of therapy may be of benefit in such anti-VEGF-resistant eyes with DME.

Some investigators have evaluated other therapeutic options not traditionally applied to retinal diseases as well, including methotrexate and interferon alpha. Methotrexate is an antimetabolite that is commonly used systemically as an immunosuppressive agent. Previously, intravitreal methotrexate has been applied as the treatment of intraocular lymphoma and proliferative vitreoretinopathy [157,158]. Interferon alpha is another commonly used systemic medication in the treatment of numerous hematologic conditions. Topical application has been used for the treatment of conjunctival malignancies and even in pterygium surgery to help prevent recurrence [159,160]. Both intravitreal methotrexate and topical interferon alpha have similar theoretical benefits in the treatment of anti-VEGF-resistant DME as both have anti-inflammatory properties similar to the steroid therapy discussed previously. Falavarjani et al. prospectively evaluated 18 anti-VEGF-resistant eyes with DME treated with a single intravitreal methotrexate injection and noted significant improvement in visual acuity at one, three and six months. There was no significant change noted in macular thickness [161]. Similarly, Maleki et al. studied five eyes with anti-VEGF-resistant DME treated with topical interferon alpha initially four times a day for two to three months with a slow taper thereafter. Macular thickness improved significantly in these eyes after one month and all eyes were noted to have stable or improved visual acuity as well [162]. These alternative anti-inflammatory therapies have only been utilized in the above limited reports, but these results appear to demonstrate a need for further research as a benefit in anti-VEGF-resistant eyes appears to exist.

## 5. Retinal Vein Occlusions

### Steroids

Similar to the case with DME, the use of intravitreal steroid therapy in the treatment of RVOs has a long history. The initial SCORE studies (Standard Care vs. Corticosteroid for Retinal Vein Occlusion) demonstrated the benefit of IVTA in the treatment of central RVOs (CRVOs) but did not report significant benefit in branch RVOs (BRVOs) [163,164]. Once again, the anti-inflammatory and anti-angiogenic capacity of steroids such as IVTA and DEX assist in the resolution of the macular edema that results from RVOs. The use of intravitreal anti-VEGF injections has largely replaced steroids as the primary treatment of RVOs, but there still exists a subset of eyes that will be resistant to such therapy.

**Figure 2:**
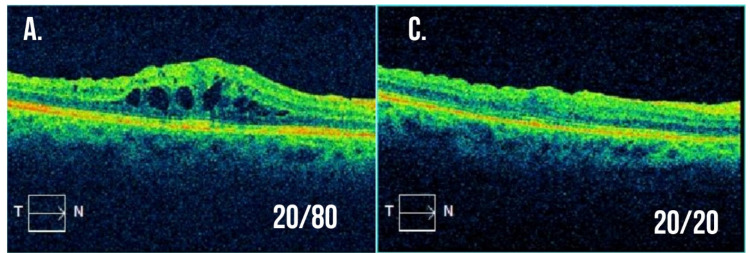

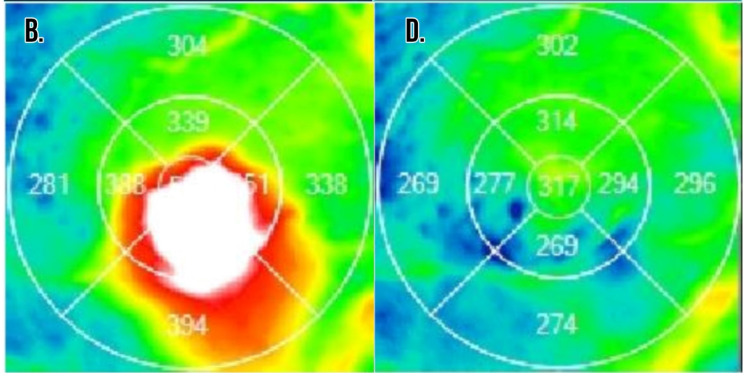
Example optical coherence tomography for a patient with branch retinal vein occlusion resistant to intravitreal anti-VEGF injections. (**A**,**B**) Macular cross-section and thickness map following multiple intravitreal anti-VEGF injections with visual acuity 20/80 and significant macular edema present. The decision was made to treat with a combination of intravitreal dexamethasone implant and anti-VEGF injection at this visit. (**C**,**D**) Macular cross-section and thickness map two months after the combination treatment with visual acuity now 20/20 and resolution of macular edema.

There has been a number of studies exploring the use of the DEX implants in the treatment of anti-VEGF-resistant RVOs demonstrating an overall benefit for such therapy. Yong et al. reported on one eye with anti-VEGF-resistant CRVO with a significant improvement noted in both visual acuity and macular thickness six months after a single DEX implant [165]. Ip et al. prospectively evaluated 14 eyes with either a CRVO or hemiretinal RVO resistant to IVA therapy switched to treatment with the DEX implant. These eyes received either one or two implants over a six-month follow-up. No significant improvement in visual acuity or macular thickness were noted in these eyes [166]. Georgalas et al. also prospectively evaluated anti-VEGF-resistant eyes with either CRVO (10 eyes) or BRVO (13 eyes) over a one-year study. Treatment was performed every six months PRN with a significant improvement in macular thickness noted in both groups at one year. Visual acuity, however, only improved significantly in the BRVO cohort with a nonsignificant improvement noted in the CRVO cohort [167]. In another retrospective study, Wolfe et al. treated 14 eyes with anti-VEGF-resistant RVOs and noted a significant improvement in both visual acuity and macular thickening. An interesting additional point noted by the authors was the correlation between an insufficient response in macular thickening on OCT (defined as ≤25% reduction in central macular thickness) one month after the first intravitreal anti-VEGF injection and long-term unresponsiveness to anti-VEGF therapy [168]. Finally, we reported our experience in a prospective study of 10 eyes with RVOs (9 with BRVO and 1 with CRVO) resistant to anti-VEGF injections treated with DEX implants every four months or PRN for 1 year. These eyes experienced significant improvements in macular thickening and on multifocal electroretinography testing, but visual acuity results were limited by cataract progression [169]. Figure 2 demonstrates the significant improvement in macular edema and visual acuity experienced in an eye with BRVO unresponsive to numerous anti-VEGF injections after a single DEX implant. Overall, eyes with BRVO resistant to anti-VEGF appear to show a substantial response to the DEX implant whereas the response to the DEX implant in eyes with CRVO is less robust.

## 6. Future Considerations—Ranibizumab Port Delivery System

The development of anti-VEGF resistance in many eyes may in part be due to diminishing levels of anti-VEGF which naturally occurs between treatments. As discussed above, some have counteracted this by decreasing treatment intervals or increasing treatment doses. On the horizon is another alternative solution to this troubling scenario, the ranibizumab port delivery system (RPDS). The RPDS is a surgically implanted refillable reservoir that continuously delivers ranibizumab to the vitreous cavity. A phase 3 clinical study is ongoing (ClinicalTrials.gov: NCT03677934), but earlier studies have demonstrated its safety with comparable visual acuity and macular thickness results to IVR therapy in the treatment of eAMD [170,171]. This technology’s capacity to continuously deliver anti-VEGF may be paramount to overcoming anti-VEGF resistance in all the previously discussed diseases.

Although targeting VEGF continues to be the mainstay of therapy, as discussed above, there are numerous pathways and targets involved in the development of macular edema in retinal diseases. Research is ongoing into investigational drugs that can utilize these alternative pathways including angiopoietin and CCR3 [172,173,174]. Such pathways could provide therapeutic options that can be used alone or in combination with anti-VEGF to potentially improve effectiveness or prolong duration of response [175]. A Better understanding of which of these pathways underlie the development of anti-VEGF resistance will also be a significant step forward in the successful treatment of this challenging situation.

## 7. Conclusions

**Figure 3:**
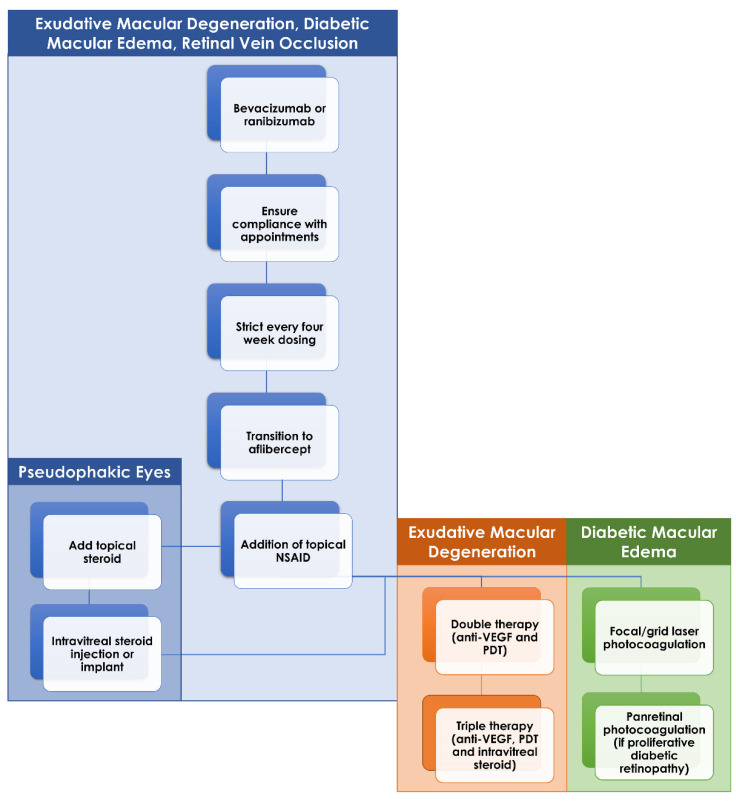
Flow chart of recommend management of anti-VEGF-resistant macular edema in exudative macular degeneration (eAMD), diabetic retinopathy and retinal vein occlusions. If initial therapy with bevacizumab or ranibizumab fails, then ensuring patient adherence to injection schedule is a key first step. Following this would be a strict injection schedule, one injection every four weeks, and transition to aflibercept if still active. Topical therapy with nonsteroidal anti-inflammatory drugs and, if pseudophakic, steroids can be initiated if the macular edema remains resistant. Intravitreal steroid injections or implants can be added in those eyes without contraindications. Finally, in eAMD and diabetic retinopathy, the addition of laser therapy (photodynamic therapy for eAMD and focal/grid laser or panretinal photocoagulation in diabetic retinopathy) can be considered in those eyes that remain resistant to all of the aforementioned treatments.

Treatment resistance to intravitreal anti-VEGF injections represents a clinical dilemma for eAMD, diabetic retinopathy and RVOs. Various alternative treatment options exist with varying degrees of success and selection of the best treatment approach must largely be determined based on the clinical scenario. An important consideration before initiating one of these alternative approaches is the patient’s adherence to scheduled injection appointments. Certain patients cannot tolerate even being a few days late for their scheduled injection so it may be necessary to first introduce a strict injection schedule, with one injection every four weeks. Following this, many patients may respond to a simple switch of anti-VEGF agents while others may require more regular injections or even injections of larger doses than utilized in standard protocols. Beyond that, the addition of supplemental intravitreal steroids either in the form of IVTA, DEX implant or FA implant appears to be of benefit most often in the retinal vascular diseases. Laser therapy (in the form of PDT in eAMD or thermal laser in diabetic retinopathy) is useful when steroid therapy fails or is contraindicated by other patient characteristics such as poorly controlled glaucoma (Figure 3). Finally, research is continuing into novel treatment options that may provide the much-needed therapeutic agent in the continued battle against these blinding conditions.

## Figures and Tables

**Table 1:** Miscellaneous Treatments of Anti-VEGF-Resistant Exudative Macular Degeneration.

Authors	Publication Year	Intervention	# of Eyes	Study Design	Findings
Lee et al. [96]	2019	Dorzolamide-Timolol	15	Interventional Case Series	Following four months of combination intravitreal anti-VEGF injections and dorzolamide-timolol found significant improvement in macular thickening, but no change in visual acuity.
Li et al. [97]	2017	Topical Nonsteroidal Anti-Inflammatory Drugs (NSAIDs)		Meta-Analysis	These studies were not evaluating anti-VEGF-resistant eyes. Six studies included utilizing either ketorolac four times a day or bromfenac twice a day along with PRN anti-VEGF injections compared to anti-VEGF monotherapy. Significantly fewer injections required with bromfenac co-treatment, significantly greater decrease in macular thickness with any NSAID and no change in visual acuity.
Zur et al. [98]	2015	Epimacular Strontium-90 Brachytherapy	22	Retrospective	No significant change in macular thickness or visual acuity one year after treatment.
Zhao et al. [99]	2019	Oral Spironolactone	21	Retrospective	Concurrently received oral spironolactone with intravitreal anti-VEGF injections with significant improvement in macular thickness that reversed with discontinuation of spironolactone. No change in visual acuity throughout study.
Peyman et al. [100]	2011	Oscillatory Transpupillary Thermotherapy	4	Case Series	One eye with visual acuity improvement 20/60 to 20/30 with unchanged visual acuity in other eyes after an average of one year of follow-up.
Shah and Haller [101]	2012	Pars Plana Vitrectomy for Vitreomacular Traction	1	Case Report	Visual acuity improved from 20/200 to 20/100.
Kimura et al. [102]	2016	Pars Plana Vitrectomy for Vitreomacular Traction or Epiretinal Membrane	6	Case Series	Significant improvement in macular thickness and improved responsiveness to intravitreal anti-VEGF injections. No change in visual acuity.
Zheng et al. [34]	2016	PDGF		Cultured Cells and Mouse Model	Upregulation of PDGF occurs with VEGF blockade which may contribute to anti-VEGF resistance. Theorize that combination treatment of VEGF and PDGF may counteract this resistance.
Zhu et al. [103]	2020	Apolipoprotein A-1 Binding Protein		Mouse Model	Cholesterol-laden macrophages tied to anti-VEGF resistance. Improved response to intravitreal anti-VEGF injections when combined with apolipoprotein A-1 binding protein which increases removal of cholesterol from macrophages to decrease its activity.
